# Cardiovascular secondary prevention in high-risk patients: a randomized controlled trial sub-study

**DOI:** 10.1186/s12872-015-0115-0

**Published:** 2015-10-14

**Authors:** Stina Jakobsson, Anna-Lotta Irewall, Fredrik Bjorklund, Thomas Mooe

**Affiliations:** Department of Public Health and Clinical Medicine, Division of Medicine, Ostersund sjukhus, Umea University, Umea, Sweden

**Keywords:** Acute coronary syndrome, Myocardial infarction, Stroke, Transient ischemic attack, Diabetes mellitus, Chronic renal insufficiency, Secondary prevention, Cardiovascular disease

## Abstract

**Background:**

Enhanced cardiovascular secondary preventive follow-up is needed to improve adherence to recommended low-density lipoprotein cholesterol (LDL-C) and blood pressure (BP) levels. Patients with diabetes mellitus (DM) or chronic kidney disease (CKD) have a high risk of recurrent events. Secondary prevention is therefore essential in these patients.

**Methods:**

Patients with acute coronary syndrome, stroke, or transient ischemic attack were randomized to nurse-based telephone follow-up (intervention) or usual care (control). LDL-C and BP were measured at 1 month (baseline) and 12 months post-discharge. Intervention patients with above-target values at baseline received medication titration to achieve treatment goals. Values measured for control patients were given to the patient’s general practitioner for assessment.

**Results:**

The final analyses included 225 intervention and 215 control patients with DM or CKD. Among patients with above-target baseline values, the following 12-month values were recorded for intervention and control patients, respectively: LDL-C, 2.2 versus 3.0 mmol/L (*p* < 0.001); and median systolic BP (SBP), 140 versus 145 mmHg (*p* = 0.26). Among patients with above-target values at baseline, 52.3 % of intervention patients reached target LDL-C values at 12 months versus 21.3 % of control patients (absolute difference of 30.9 %, 95 % CI 16.1 to 43.8 %), and there was a non-significant trend of more intervention patients reaching target SBP (49.4 % versus 36.8 %; absolute difference of 12.6 %, 95 % CI −1.7 to 26.2 %).

**Conclusions:**

Cardiovascular secondary prevention with nurse-based telephone follow-up was more effective than usual care in improving LDL-C levels 12 months after discharge for patients with DM or CKD.

**Trial registration:**

ISRCTN registry; ISRCTN96595458 (date of registration 10 July 2011) and ISRCTN23868518 (date of registration 13 May 2012).

**Electronic supplementary material:**

The online version of this article (doi:10.1186/s12872-015-0115-0) contains supplementary material, which is available to authorized users.

## Background

Following an acute coronary syndrome (ACS) or stroke there is a high risk of recurrent events. A population based study of patients with ischemic stroke found that the cumulative five year risk of MI, stroke or vascular event was 29 % [1]. Furthermore, data from Swedish Web-system for Enhancement and Development of Evidence-based care in Heart disease Evaluated According to Recommended Therapies (SWEDEHEART) show that 13 % of the patients are readmitted for a cardiac disease during the year following a myocardial infarction and 4 % suffer an ischemic stroke within one year [2, 3]. Morbidity and case fatality after an ACS or stroke can be reduced by secondary preventive treatments that lower lipid levels and blood pressure (BP) [4]. However, implementation of such treatments has been unexpectedly difficult, with low proportions of patients reaching the recommended blood lipid levels and BP [5–7]. In EUROASPIRE IV patients with an ACS or who were revascularized with percutaneous coronary intervention or coronary artery bypass grafting were examined between six months and three years after the qualifying event. Approximately 80–90 % of the patients received a blood pressure lowering drug and a statin, but of those receiving treatment only 57 and 21 % reached target levels for BP and low-density lipoprotein cholesterol (LDL-C), respectively [7]. Various organizational strategies have been evaluated for improving secondary preventive care in patients with established cardiovascular disease (CVD), including patient education, physician education, pre-booked doctor appointments, etc. However, most investigated strategies have not effectively reduced risk factors [8, 9]. Some data suggest that telephone-based follow-up improves risk factor control [10].

Diabetes mellitus (DM) and chronic kidney disease (CKD) are common comorbidities in patients with established CVD. In a population of Medicare beneficiaries of 65 years of age or older in the United States, DM was found in approximately 40 % of the patients with ischemic heart disease or stroke and CKD was found in 30–35 % of the patients [11]. Moreover, patients with DM or CKD have a worse prognosis after a CVD. For example, one study showed that the hazard ratio of death for DM patients compared to non-DM patients one year after an ACS was increased (1.33; 95 % confidence interval 1.20–1.48) and a pooled analysis of community-based cohorts showed that over 60 % of the patients with established CVD and CKD suffered a MI, fatal coronary heart disease, stroke, or died during a 10-year time period, compared to approximately 35 % of the non-CKD patients [12, 13]. While the age-standardized death rates after an ACS or stroke have decreased over recent years [14], the prevalences of DM and CKD are increasing [15, 16]. Thus, there is a potentially growing population with CVD in combination with DM and/or CKD. These patients have a very high risk of recurrent events and secondary prevention is therefore essential.

The Nurse-Based Age-Independent Intervention to Limit Evolution of Disease (NAILED) ACS and NAILED stroke risk factor trials are ongoing randomized controlled trials that aim to improve secondary prevention after ACS, stroke, and transient ischemic attack (TIA) through nurse-based telephone follow-up of modifiable risk factors. Considering the importance of secondary prevention, we performed the present study to investigate the effects of follow-up according to the NAILED trial in a high-risk patient population with DM or CKD. We hypothesized that compared to usual care, the NAILED follow-up would more effectively improve LDL-C levels and BP at 12 months after an ACS, stroke, or TIA.

## Method

The rationale and the open, population-based, randomized controlled design of the NAILED trials have been previously described [17, 18]. In brief, all patients hospitalized at Ostersund Hospital with a diagnosis of myocardial infarction, unstable angina, stroke, or TIA were assessed for inclusion. Ostersund Hospital is the only hospital in the county of Jamtland, Sweden, and has a rural catchment area with a population of approximately 125,000 inhabitants. Study nurses identified eligible patients with the physical and mental capacity to communicate by telephone. Patients suffering from deafness, aphasia, dementia, or severe (often terminal) diseases were not included. Participants in other ongoing trials were also excluded. All eligible patients were informed about the study and asked to sign a written informed consent. Those who agreed to participate were randomized to intervention or control in a 1:1 ratio. The random allocation sequence was computer generated in blocks of four and stratified for sex and type of ACS (unstable angina or myocardial infarction) for ACS patients. For patients with stroke or TIA the random sequence was generated in blocks of four and was stratified for sex and for degree of disability (modified Rankin Scale <3 or ≥3).

This study was approved by the Regional Ethics Committee, Umea University 28 October 2009 (reference number Dnr-09-142M).

Patient characteristics, including medical history, were recorded during the initial hospitalization. At 1 month after discharge, baseline measurements of blood lipids and BP were performed by a health care professional at the patients’ closest health care facility and reported to the study team. Corresponding follow-up measurements were performed at 12 months after discharge. Shortly after the measurements of blood lipids and BP, a study nurse contacted participants in both study groups by telephone and interviewed them about their well-being and adherence to medical treatment.

In the control group, LDL-C and BP values were forwarded directly to the patient’s general practitioner (GP) for assessment, without further action from the study team. The patients in the control group received secondary preventive care according to local standard management. Secondary preventive treatment was generally initiated in-hospital and thereafter, the patients’ GPs held primary responsibility for their care. The standard of care for DM patients is, according to local guidelines, a yearly visit to their GP and a yearly visit to a nurse. During the visits a risk factor assessment and intervention regarding established risk factors for CVD is performed. The secondary preventive management of CKD patients does not differ from the overall CVD population. Patients in both study groups could at any time book an appointment with their GP.

For patients randomized to intervention, if they had not reached the target LDL-C level and/or BP their medication was titrated according to guidelines. The initial target value for LDL-C level was <2.5 mmol/L. On 31 March, 2013, the local guidelines for diabetic patients changed such that the target level was <1.8 mmol/L. Throughout the trial period, the target BP value was <140/90 mmHg. Tests were repeated within approximately 4 weeks. If necessary, medication was further titrated until target values were reached or no further changes were considered appropriate. Decisions regarding titration and medication were made by a study physician.

The present analysis included patients with DM or CKD, who were admitted between 1 January, 2010 and 30 June, 2013 and who received a 12-month follow-up call. Patients were considered to have DM if they received glucose-lowering medication or dietary treatment at discharge from their initial hospitalization. During hospitalization, a creatinine value was registered and an estimated glomerular filtration rate (eGFR) was calculated using the Chronic Kidney Disease Epidemiology Collaboration (CKD-EPI) creatinine equations [19]. Patients were assumed to be Caucasian. An eGFR < 60 mL/min/1.73 m^2^ was considered to indicate the presence of CKD [20].

### Outcomes

The primary outcome in the present analysis was to determine the difference in LDL-C and systolic blood pressure (SBP) between patients in the two groups at 12 months follow-up, before any medication titrations. The differences in diastolic blood pressure (DBP) and proportions of reached target values between the groups were secondary outcomes. Exploratory data analyses with between-group comparison of risk factor values and proportion of patients within target values were performed separately for patients with DM and CKD.

### Availability of data

Deposition of patient level data in a public repository was not specified in the study protocol as approved by the ethics committee before the study started. Patient level data will be available on request, provided that an approval from the Regional Ethics Committee is given.

### Statistics

The results are presented as median and percentiles for continuous variables, and as percentages for categorical variables. To analyse the effects of the intervention, risk factor values and the proportion of achieved target values at 12 months were analysed separately among patients who did and did not meet risk factor target values at baseline. To assess between-group differences, the Mann–Whitney *U*-test was used for continuous variables and Pearson’s *χ*^2^ test for categorical variables. Within-group changes from baseline to 12-month follow-up were examined using the Wilcoxon signed-rank test.

Power analysis showed that study groups of approximately 80 participants were required to detect between-group differences of 0.4 mmol/L in mean LDL-C, of 8 mmHg in mean SBP, and of 20 % in the proportions of reached target values (alpha 0.05, two-tailed, power 80 %). Subjects with missing values were excluded from the analysis. The Bonferroni technique was used to adjust the alpha value because multiple comparisons were performed within each group. After adjustment an alpha value of 0.01 was considered significant in the risk factor value analyses. Statistical analyses were performed using SPSS 20.0 software.

## Results

**Fig. 1:**
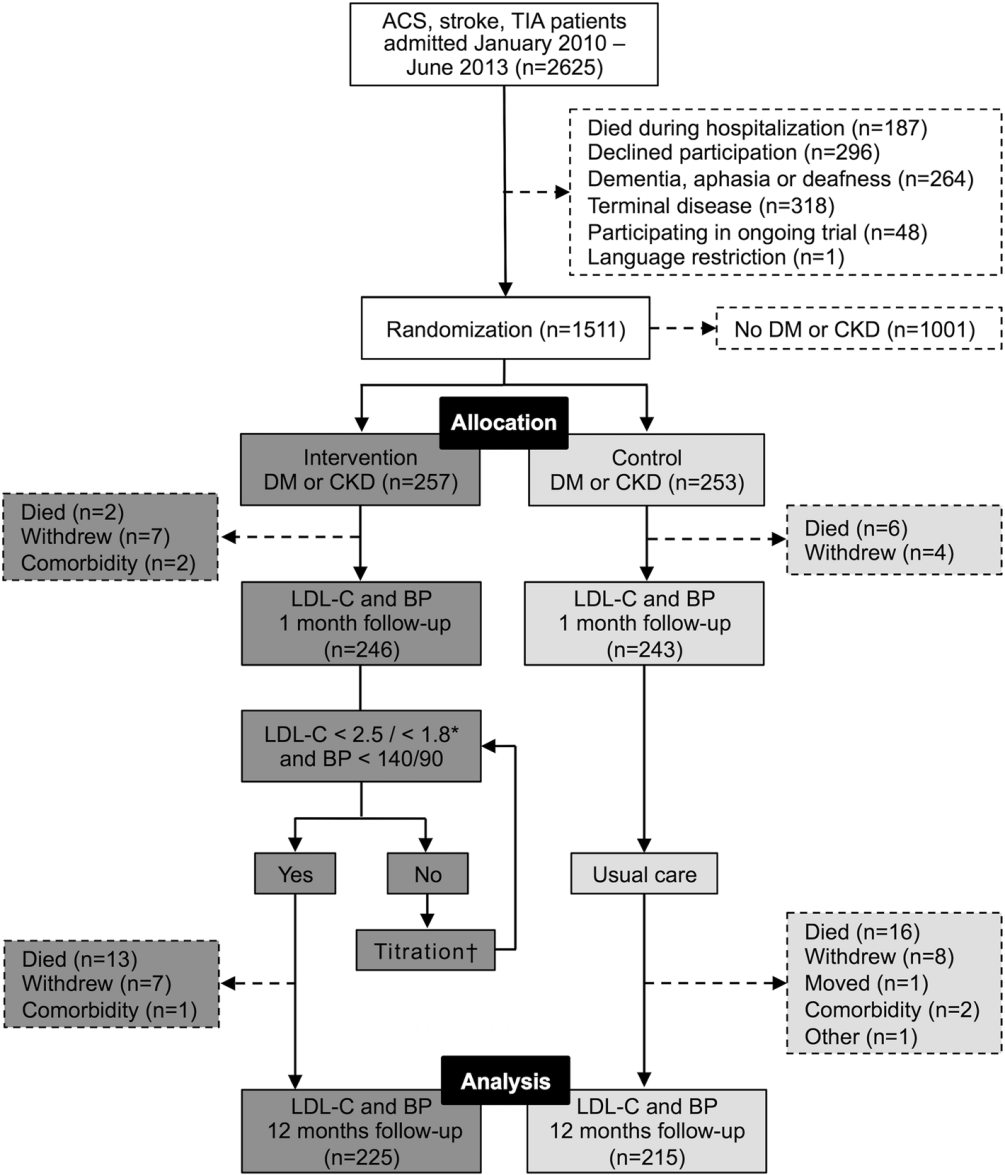
Study flow diagram. *The target LDL-C value was <2.5 mmol/L until 31 March, 2013, when local guidelines for diabetic patients changed this target to <1.8 mmol/L; †Medication was titrated until target values were reached or no further changes were considered appropriate. *n*: number of cases; DM: diabetes mellitus; CKD: chronic kidney disease; LDL-C: low-density lipoprotein cholesterol; BP: blood pressure

Between January 2010 and June 2013, 1511 ACS, stroke, or TIA patients were included in the study. Of these subjects, 758 (33.9 % [257/758] with DM or CKD) were randomized to the intervention group and 753 (33.6 % [253/753] with DM or CKD) to the control group. The final analyses included 225 patients with DM or CKD in the intervention group and 215 patients in the control group (Fig. 1). Table 1 shows the patients’ in-hospital characteristics. The groups were well matched except for that fewer patients in the intervention group received lipid-lowering treatment at admission (40.9 % versus 52.6 %). Thirty-five patients in the intervention group and 36 patients in the control group had both DM and CKD.

**Table 1:** In-hospital patient characteristics

	Intervention	Control
	*n* = 225	*n* = 215
Age in years, median (25th–75th percentile)	75 (67–82)	75 (67–80)
Female, *n* (%)	86 (38.2)	75 (34.9)
Smoking, *n* (%)	25 (11.1)	23 (10.7)
BMI, median (25th–75th percentile)	27.9 (24.7–31.0)	27.2 (24.6–30.2)
Diabetes mellitus, *n* (%)	139 (61.8)	128 (59.5)
Chronic kidney disease, *n* (%)	121 (53.8)	123 (57.2)
Prior disease, *n* (%)		
Coronary artery disease	52 (23.1)	57 (26.5)
Stroke/TIA	52 (23.1)	44 (20.5)
Peripheral artery disease	8 (3.6)	9 (4.2)
Qualifying event, *n* (%)		
Unstable angina	7 (3.1)	9 (4.2)
NSTEMI	82 (36.4)	70 (32.6)
STEMI	30 (13.3)	24 (11.2)
TIA	35 (15.6)	42 (19.5)
Ischemic stroke	68 (30.2)	66 (30.7)
Haemorrhagic stroke	3 (1.3)	4 (1.9)
Treatment at admission, *n* (%)		
Antihypertensive drug (≥1)	183 (81.3)	188 (87.4)
Lipid-lowering drug	92 (40.9)	113 (52.6)
Treatment at discharge, *n* (%)		
Antihypertensive drug (≥1)	211 (93.8)	199 (92.6)
Lipid-lowering drug	185 (82.2)	180 (83.7)

*n* number of cases, *BMI* body mass index (calculated as weight in kilograms divided by the squared height in meters), *TIA* transient ischemic attack, *NSTEMI* non-ST elevation myocardial infarction, *STEMI* ST elevation myocardial infarction

### Risk factor values at 12 months

The baseline median values for LDL-C, SBP, and DBP, for patients not meeting target values were similar between the intervention and control groups (Table 2). At the 12-month follow-up, both groups showed significant decreases in these risk factor values compared to baseline. LDL-C levels at 12 months were significantly lower in the intervention group (2.2 mmol/L) compared to the control group (3.0 mmol/L) (*p* < 0.001). We observed a non-significant trend towards a lower SBP value in the intervention group (140 mmHg) compared to in the control group (145 mmHg) (*p* = 0.26) (Table 2). Additional file 1 shows the corresponding data for the patients who met target values at baseline. Among these patients, the risk factor values generally increased between baseline and 12 months and there were no significant between-group differences.

**Table 2:** Risk factor values for patients who had above-target values at baseline

	Intervention	Control	*p*-value
LDL-C ≥ 2.5/1.8^a^ baseline, n	87	78	
LDL-C baseline, median (25th–75th percentile)	2.9 (2.6–3.5)	2.9 (2.7–3.7)	0.74
LDL-C 12 months, median (25th–75th percentile)	2.2 (1.8–3.0)***	3.0 (2.4–3.4)**	<0.001
SBP ≥ 140 baseline, n	89	95	
SBP baseline, median (25th–75th percentile)	150 (142–160)	150 (140–160)	0.97
SBP 12 months, median (25th–75th percentile)	140 (129–155)***	145 (130–158)***	0.26
DBP ≥ 90 baseline, n	25	26	
DBP baseline, median (25th–75th percentile)	90 (90–95)	90 (90–96)	0.71
DBP 12 months, median (25th–75th percentile)	80 (75–86)***	80 (75–91)***	0.48

**p ≤ 0.01, ***p ≤ 0.001, indicating a significant change of median values within each group between baseline and 12 months; ^a^ The target LDL-C value was <2.5 mmol/L until 31 March, 2013, when local guidelines for diabetic patients changed this target to <1.8 mmol/L. *LDL-C* low-density lipoprotein cholesterol (mmol/L), *n* number of cases, *SBP* systolic blood pressure (mmHg), *DBP* diastolic blood pressure (mmHg)

### Proportions of patients who achieved target values at 12 months

**Fig. 2:**
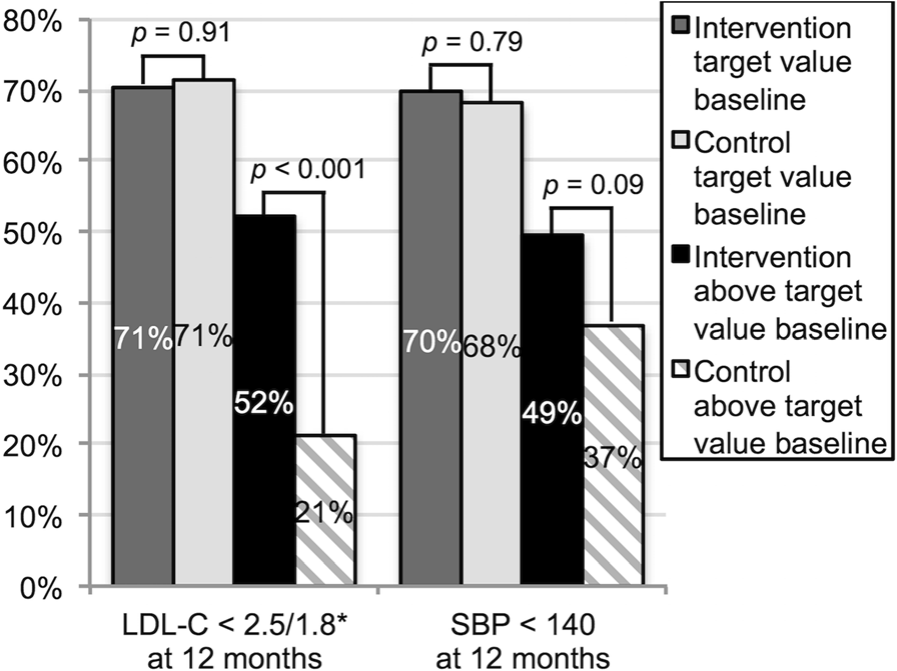
Proportion of patients who reached target values at 12 months, stratified by whether the patients were within or above target values at baseline. *The target LDL-C value was <2.5 mmol/L until 31 March 2013, when local guidelines for diabetic patients changed this target to <1.8 mmol/L. SBP: systolic blood pressure; LDL-C: low-density lipoprotein cholesterol

At baseline, the target LDL-C level was met by 60.8 % (135/222) in the intervention group and by 62.7 % (131/209) of the control group (absolute difference of 1.9 %, 95 % CI −7.2 to 10.9 %), target SBP by 60.3 % (135/224) of the intervention group and 55.8 % (120/215) of controls (absolute difference of 4.5 %, 95 % CI −4.7 to 13.6 %), and target DBP by 88.8 % (199/224) of the intervention group and 87.9 % (189/215) of controls (absolute difference of 0.9 %, 95 % CI −5.1 to 7.1 %). Figure 2 shows the proportions of patients who reached target LDL-C and SBP values at 12 months stratified by baseline values (reaching target or not). Among patients with above-target values at baseline, a significantly higher proportion of the intervention group showed target LDL-C values at 12 months compared to controls (52.3 % [45/86] versus 21.3 % [16/75]; absolute difference of 30.9 %, 95 % CI 16.1 to 43.8 %). The corresponding proportions of target SBP values at 12 months for patients with above target at baseline were 49.4 % (44/89) and 36.8 % (35/95) (absolute difference of 12.6 %, 95 % CI −1.7 to 26.2 %), respectively.

**Fig. 3:**
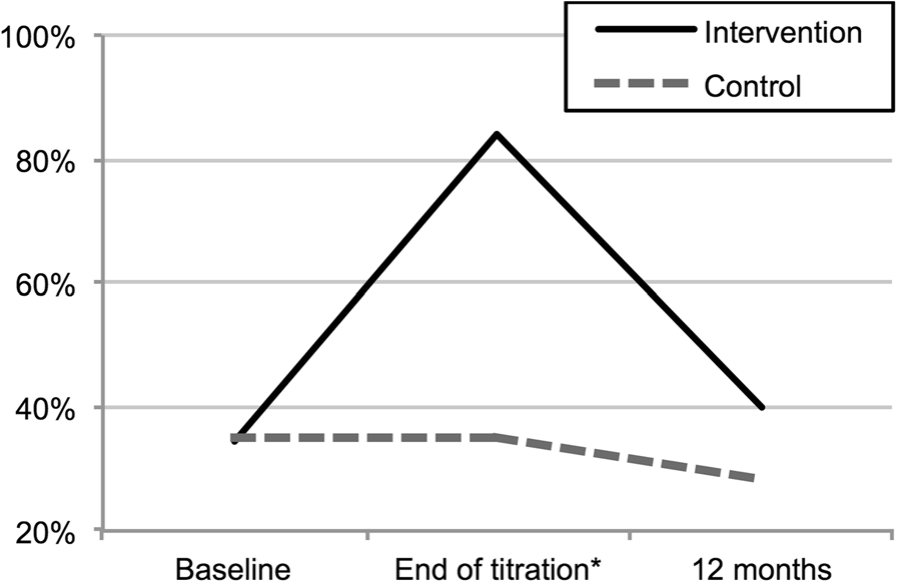
Proportion of patients reaching target LDL-C, SBP and DBP. *The proportion reported for control patients is the same as at baseline since control patients did not receive any medication titration within the study period

Figure 3 shows the proportion of patients who met all target values (LDL-C, SBP and DBP) at baseline, after medication titration, and at 12 months. At baseline this proportion did not differ between the groups (intervention 34.2 % [76/222] versus control 35.1 % [74/211]; absolute difference of 0.8 %, 95 % CI −8.1 to 9.8 %). After medication titration, 83.8 % (186/222) of the patients in the intervention group reached all target values (including patients who already showed target values at baseline) (absolute difference of 48.7 %, 95 % CI 40.1 to 56.2 %). At 12 months 40.0 % (88/220) of the intervention patients and 28.2 % (59/209) of the control patients met all target values (absolute difference of 11.8 %, 95 % CI 2.8 to 20.5 %).

### Sub-group analysis

Repeating the analyses separately for patients with DM and CKD produced results that were largely similar to the combined analyses with regard to risk factor value differences between intervention and control patients (see Additional files 2 and 3). The separate analyses showed that 43.5 % (20/46) of DM patients in the intervention group whose LDL-C levels were above target at baseline had reached target LDL-C levels at 12 months, compared to 26.5 % (9/34) in the control group (absolute difference of 17.0 %, 95 % CI −4.3 to 35.6 %). The corresponding results in the CKD group were 60.4 % (32/53) versus 14.3 % (7/49), respectively (absolute difference of 46.1 %, 95 % CI 27.8 to 60.1 %).

Among the CKD patients in the intervention group who showed above-target SBP at baseline, separate analysis showed that 59.6 % had reached target at 12 months, while combined DM and CKD analysis showed a lower proportion (49.4 %). Thus, more CKD intervention patients reached target SBP at 12 months compared to CKD control patients (59.6 % [28/47] versus 35.2 % [19/54]; absolute difference of 24.3 %, 95 % CI 4.9 to 41.5 %).

## Discussion

The present results demonstrated that a structured, secondary preventive, nurse-based telephone follow-up of modifiable risk factors after an ACS, stroke, or TIA led to more prevalent LDL-C level improvement over 12 months among high-risk patients with DM or CKD as compared to the impact of standard care. We also observed a more prevalent decrease of SBP in the intervention group compared to the control group, but this difference was not statistically significant—possibly because the patient groups were too small. A between-group difference of 5 mmHg is probably of clinical significance; however, the power of the study was only sufficient to detect an 8 mmHg difference. With regard to the proportion of patients reaching target values, the examined intervention was significantly more effective concerning LDL-C, and showed a trend of being more effective for SBP.

Previous population-based studies have shown that approximately 15–60 % of participants reach target values for LDL-C or BP [5–7]. In the present study, as many as 83.8 % of the patients in the intervention group reached the target LDL-C, SBP, and DBP values after medication titration (Fig. 3). At 12 months, the proportion of participants with target values had decreased to 40 %. Similarly, a fairly large proportion of the patients with target levels at baseline showed above-target values at 12 months. This might be explained by decreased medication adherence over time. More frequent follow-ups during the first year are probably beneficial, but our present findings indicate that secondary prevention efforts should extend beyond 12 months of follow-up. The NAILED trials are planned to follow-up patients for a minimum of 36 months [17, 18].

Our results are in agreement with a previous meta-analysis of 11 studies, which found that telehealth interventions (phone and internet communication between patient and healthcare providers) effectively reduced risk factors among patients with coronary heart disease [10]. The meta-analysis showed that intervention decreased the weighted mean difference for LDL-C by 0.41 mmol/L and for SBP by 4.69 mmHg. This can be compared to the presently observed median decrease of 0.8 mmol/L in LDL-C and of 5 mmHg in SBP. The present study showed a larger risk factor reduction, even though the median patient age was 75 years compared to the mean age of 57–64 years in the meta-analysis [10]. The age of the NAILED study population is more representative of the general ACS, stroke, and TIA population than the younger patients included in previous trials evaluating telephone-supported secondary preventive follow-up.

We believe that prompt medication titration and follow-up every four weeks until target values are reached represent a key component of the NAILED method. Two recently published Cochrane reviews evaluate organizational strategies to improve risk factor control after a CVD, and report only weak effects or no effect on modifiable risk factor levels. None of the included studies that are available in English used a method involving prompt medication titration [8, 9]. In the PREVENTION study, prompt medication titration performed by pharmacists authorized to prescribe medication resulted in increased proportions of patients reaching the desired risk factor levels [21].

Our separate analyses of DM or CKD patients showed that the CKD patients seemed to gain more from the NAILED intervention. This may be partly explained by the fact that DM patients are carefully monitored within the Swedish healthcare system. In the county of Jamtland, DM patients have a standard yearly visit to the GP and a yearly visit to a nurse for evaluation of the patients’ diabetes-associated problems, such as BP and LDL. Consequently, their risk factors are typically better monitored compared to the general CVD population.

The greater benefit seen in the intervention patients with CKD may also be due to the concerns that GPs have regarding medication usage in patients with decreased kidney function, which may make them hesitant to intensify treatment. A meta-analysis investigating statin treatment in CKD patients reported that statins had beneficial effects on mortality and cardiovascular events in pre-dialysis CKD patients, without increased adverse effects [22]. Patients with terminal disease were not included in the NAILED trials. Thus, it is unclear why 85.7 % of the control CKD patients who were above target LDL-C levels at baseline were still above target at 12 months.

### Limitations

The method used in the NAILED trial was designed to be broadly implementable in clinical practice and, therefore, all patients were considered for inclusion if they were able to participate and were not enrolled in a concurrent trial. A patient’s ability to participate was assessed by a study nurse, introducing a risk of arbitrariness in the decision of eligibility. Another potential weakness was the BP measurement procedure, which was performed by a large number of healthcare professionals at different healthcare centres in the county and using different devices. The letter of referral included simple instructions for what was considered a standardized BP measurement [17, 18], but the accuracy of the individual BP measurements could have varied. However, this should not be a major concern, since it is reasonable to believe that any inaccuracy would have been fairly equally distributed between the two study groups. Finally, we defined CKD according to an estimate from a single creatinine value. This differs from the recommended definition of CKD, which requires two measurements taken three months apart [20].

## Conclusion

Our present results showed that nurse-based telephone follow-up including medication titration was more effective than usual care for reducing LDL-C levels and increasing the proportion of patients showing target LDL-C values at 12 months after an ACS, stroke, or TIA among high-risk patients with DM or CKD. The corresponding decrease in SBP and increase in the proportion of patients with target SBP values at 12 months did not significantly differ from those seen in patients with usual care.

The results support our hypothesis that the NAILED follow-up is more efficient than usual care to reduce levels of modifiable risk factors after a CVD in patients with DM or CKD. Future reports will reveal whether nurse-based telephone follow-up is more effective than usual care to achieve long-term risk factor control.

## Additional files

Additional file 1:
**Risk factor values for patients who had target values at baseline.** (PDF 68 kb) Additional file 2:
**Risk factor values for DM patients who had above-target values at baseline.** (PDF 75 kb) Additional file 3:
**Risk factor values for CKD patients who had above-target values at baseline.** (PDF 70 kb)
