# Supplementary material for: Cardiovascular secondary prevention in high-risk patients: a randomized controlled trial sub-study
Source: BMC Cardiovasc Disord. 2015 Oct 14;15:125. doi: 10.1186/s12872-015-0115-0 (PMC4607173; doi:10.1186/s12872-015-0115-0)
Supplement: Additional file 1: — Risk factor values for patients who had target values at baseline. (PDF 68 kb) [file 12872_2015_115_MOESM1_ESM.pdf]

Additional file 1. Risk factor values for patients who had target values at baseline

	Intervention	Control	*p*-value
**LDL-C <2.5/1.8† baseline, n**	135	131	
**LDL-C baseline, median (25th–75th percentile)**	1.8 (1.5-2.1)	1.9 (1.5-2.1)	0.77
**LDL-C 12 months, median (25th–75th percentile)**	2.0 (1.6-2.3)***	1.9 (1.5-2.4)***	0.76
**SBP <140 baseline, n**	135	120	
**SBP baseline, median (25th–75th percentile)**	123 (118-130)	125 (120-130)	0.66
**SBP 12 months, median (25th–75th percentile)**	130 (120-140)***	130 (118-142)***	0.62
**DBP <90 baseline, n**	199	189	
**DBP baseline, median (25th–75th percentile)**	75 (68-80)	74 (70-80)	0.88
**DBP 12 months, median (25th–75th percentile)**	74 (67-80)^{NS}	76 (69-81)**	0.22

**p ≤ 0.01, ***p ≤ 0.001, indicating a significant change of median values within each group between baseline and 12 months; ; †The target LDL-C value was <2.5 mmol/L until 31 March, 2013, when local guidelines for diabetic patients changed this target to <1.8 mmol/L. LDL-C: low density lipoprotein cholesterol (mmol/L); n: number of valid cases; SBP: systolic blood pressure (mmHg); DBP: diastolic blood pressure (mmHg); NS: non-significant.
