# Supplementary material for: Cardiovascular secondary prevention in high-risk patients: a randomized controlled trial sub-study
Source: BMC Cardiovasc Disord. 2015 Oct 14;15:125. doi: 10.1186/s12872-015-0115-0 (PMC4607173; doi:10.1186/s12872-015-0115-0)
Supplement: Additional file 2: — Risk factor values for DM patients who had above-target values at baseline. (PDF 75 kb) [file 12872_2015_115_MOESM2_ESM.pdf]

Additional file 2. Risk factor values for DM patients who had above-target values at baseline

	**Intervention**	**Control**	**p-value**
**LDL-C ≥2.5/1.8† baseline, n**	46	34	
**LDL-C baseline, median (25th–75th percentile)**	2.9 (2.5-3.5)	2.8 (2.7-3.6)	0.71
**LDL-C 12 months, median (25th–75th percentile)**	2.2 (1.9-3.1)***	2.7 (2.1-3.3)^{NS}	0.04
**SBP ≥140 baseline, n**	57	57	
**SBP baseline, median (25th–75th percentile)**	150 (142-160)	150 (140-160)	0.71
**SBP 12 months, median (25th–75th percentile)**	142 (130-160)***	145 (130-157)**	0.96
**DBP ≥90 baseline, n**	15	14	
**DBP baseline, median (25th–75th percentile)**	90 (90-93)	90 (90-100)	0.40
**DBP 12 months, median (25th–75th percentile)**	85 (78-89)**	80 (75-85)**	0.38

**$p \leq 0.01$, ***$p \leq 0.001$, indicating a significant change of median values within each group between baseline and 12 months; ; †The target LDL-C value was <2.5 mmol/L until 31 March, 2013, when local guidelines for diabetic patients changed this target to <1.8 mmol/L. LDL-C: low density lipoprotein cholesterol (mmol/L); n: number of valid cases; SBP: systolic blood pressure (mmHg); DBP: diastolic blood pressure (mmHg); NS: non-significant.
