# Supplementary material for: Cardiovascular secondary prevention in high-risk patients: a randomized controlled trial sub-study
Source: BMC Cardiovasc Disord. 2015 Oct 14;15:125. doi: 10.1186/s12872-015-0115-0 (PMC4607173; doi:10.1186/s12872-015-0115-0)
Supplement: Additional file 3: — Risk factor values for CKD patients who had above-target values at baseline. (PDF 70 kb) [file 12872_2015_115_MOESM3_ESM.pdf]

Additional file 3. Risk factor values for CKD patients who had above-target values at baseline

	Intervention	Control	p-value
**LDL-C ≥2.5/1.8† baseline, n**	54	52	
**LDL-C baseline, median (25th–75th percentile)**	3.0 (2.7-3.5)	3.1 (2.7-3.7)	0.33
**LDL-C 12 months, median (25th–75th percentile)**	2.2 (1.8-2.8)***	3.0 (2.6-3.7)^{NS}	<0.001
**SBP ≥140 baseline, n**	47	54	
**SBP baseline, median (25th–75th percentile)**	150 (144-155)	150 (140-160)	0.96
**SBP 12 months, median (25th–75th percentile)**	132 (126-150)***	145 (130-160)^{NS}	0.05
**DBP ≥90 baseline, n**	12	14	
**DBP baseline, median (25th–75th percentile)**	92 (90-99)	90 (90-94)	0.37
**DBP 12 months, median (25th–75th percentile)**	80 (71-84)**	84 (79-94)**	0.07

**p ≤ 0.01, ***p ≤ 0.001, indicating a significant change of median values within each group between baseline and 12 months; ; †The target LDL-C value was <2.5 mmol/L until 31 March, 2013, when local guidelines for diabetic patients changed this target to <1.8 mmol/L. LDL-C: low density lipoprotein cholesterol (mmol/L); n: number of valid cases; SBP: systolic blood pressure (mmHg); DBP: diastolic blood pressure (mmHg); NS: non-significant.
